# Supplementary material for: Content-rich biological network constructed by mining PubMed abstracts
Source: BMC Bioinformatics. 2004 Oct 8;5:147. doi: 10.1186/1471-2105-5-147 (PMC528731; doi:10.1186/1471-2105-5-147)
Supplement: Additional File 2 — The original results of the above study (non-essential files are deleted to keep the file size under the limit set by BMC bioinformatics). [file 1471-2105-5-147-S2.bz2 › chilibotAdditionalFile2/dip30/234ID8006584E758/html/IL2RB_IL2RA.html]

 


 **IL2RB** and **IL2RA** 
  
Found 1183 abstracts in PubMed, retrieved 30.  
 

 What does Google say? 
 PDF only 
| .edu only 

---

**Interactive relationship** (e.g. stimulation, inhibition, etc)

**Non-interactive relationship** (e.g. studied together, co-existance, homology, etc.)

- However, eosinophils with sparse granulation, positivity for CD25  [ **IL2RA** ] , elevated serum levels of soluble IL 2 receptor  [ **IL2RB** ] , and elevated serum levels of eosinophil cationic protein suggested activation of eosinophils.  Ref: 12462027 Rinsho Ketsueki, 2002
- Cell surface expression of CD25  [ **IL2RA** ] , CD122  [ **IL2RB** ] , CD132, and CD69 were also determined and no statistical differences were found between both groups.  Ref: 12635575 Allergy Asthma Proc< MedlineTA>Allergy Asthma Proc, 2002
- Cells stained with Annexin V Anx and antibodies to CD56, CD3, CD95, CD25  [ **IL2RA** ] , CD122  [ **IL2RB** ]  or CD132 were examined by flow cytometry.  Ref: 12594840 Eur J Immunol, 2003
- CONCLUSION The monoclonal antibody against IL 2 receptor  [ **IL2RB** ]  alpha chain CD 25  [ **IL2RA** ]  has a marked effect upon severe steroid refractory GVHD.  Ref: 12812665 Zhonghua Yi Xue Za Zhi, 2003
- Most of the surface membrane antigens CD5, CD11c, CD19, CD20, CD21, CD22, CD23, CD25  [ **IL2RA** ] , CD40, CD45, VD79b, CD80, CD95, CD122  [ **IL2RB** ] , CD124, CD126, CD130, CD154, IgM, and IgD showed a similar expression pattern in both UM CLL and M CLL patients.  Ref: 12631259 Eur J Haematol, 2003
- We observed that eosinophils from most patients with hypereosinophilia express the alpha chain of the IL 2 receptor  [ **IL2RB** ]  CD25  [ **IL2RA** ] ,.  Ref: 12672048 Eur J Immunol, 2003
- Parameters of cellular and humoral immunity differential blood count, T cell markers CD2, CD3, CD4, and CD8, B cell markers CD19 and CD20, monocyte markers CD13 and CD14, NK cell marker CD16, activation markers CD25  [ **IL2RA** ] , CD26, CD69 and HLA DR, and cytokines IL 1 receptor antagonist IL 1RA, IL 2, soluble IL 2 receptor  [ **IL2RB** ]  sIL 2R, IL 6, IL 10, and TGFbeta were measured in peripheral venous blood.  Ref: 12779014 Folia Biol Praha , 2003
- Like living Trypanosoma cruzi, its AGC10 membrane glycoprotein inhibits interleukin 2 IL 2 secretion and membrane expression of CD25  [ **IL2RA** ] , CD122  [ **IL2RB** ] , and CD132 the components of the high affinity IL 2 receptor  [ **IL2RB** ]  by activated human lymphocytes.  Ref: 12467977 Mol Biochem Parasitol< MedlineTA>Mol Biochem Parasitol, 2003
- Murine studies have suggested that a population of CD4 T cells expressing the alpha chain of the interleukin IL 2 receptor  [ **IL2RB** ]  CD25  [ **IL2RA** ]  are phenotypically anergic in response to T cell receptor stimulation and can suppress the function of CD4 and CD8 T cells.  Ref: 12514432 J Immunother< MedlineTA>J Immunother, 2003
- IL 2 was not detected in cell culture supernatants, but IL 2 receptor  [ **IL2RB** ]  alpha chain CD25  [ **IL2RA** ]  was detected in low density on a small proportion of T cells activated by C1q bearing immune complexes.  Ref: 12519387 Clin Exp Immunol, 2003
- The inhibitory effect on human T cells was assessed and demonstrated at the level of proliferation DNA synthesis, IL 2 secretion, and IL 2 receptor  [ **IL2RB** ]  CD25  [ **IL2RA** ]  upregulation.  Ref: 12835898 Inflamm Res, 2003
- Prior to testing this strategy in patients, our goal was to develop a clinical scale SD process, which involves co culture of donor lymphocytes and irradiated recipient cells, followed by the addition of an immunotoxin IT directed against the alpha chain of the IL 2 receptor  [ **IL2RB** ]  CD25  [ **IL2RA** ] , expressed on activated donor T cells.  Ref: 12473206 Cytotherapy, 2002
- Most investigated Treg cells are CD4 pos cells expressing the alpha chain of the IL 2 receptor  [ **IL2RB** ]  CD25  [ **IL2RA** ] .  Ref: 12848995 Autoimmun Rev, 2002
- BACKGROUNDTo define whether immunosuppressive agents that block the interleukin IL 2 pathway could prevent activation induced cell death of activated T cells in the graft, we measured expression of IL 2, IL 2 receptor  [ **IL2RB** ]  alpha chain CD25  [ **IL2RA** ] , IL 15, Fas, and Fas ligand by real time reverse transcription polymerase chain reaction in cardiac allografts.METHODSWe characterized the phenotype of the infiltrating cells CD3, CD68, CD25  [ **IL2RA** ]  by immunohistochemistry.  Ref: 12777860 Transplantation, 2003
- When we compared mean nocturnal levels, CD16 and CD25  [ **IL2RA** ]  T and B activated lymphocytes with expression of the a chain of IL 2 receptor  [ **IL2RB** ]  levels were higher, while CD8, CD8bright, CD20 total B cells, TcRd1 epitope of the constant domain of d chain of T cell receptor 1 and dTcS1 epitope of the variable domain of d chain of T cell receptor1 levels were lower in cancer patients.  Ref: 12797908 Int J Immunopathol Pharmacol< MedlineTA>Int J Immunopathol Pharmacol, 2003
- Supporting this view were the substantially diminished levels of IL 2, CD25  [ **IL2RA** ] , CD122  [ **IL2RB** ] , and CD132 mRNA found in AGC10 containing cultures of PHA activated lymphocytes.  Ref: 12467977 Mol Biochem Parasitol< MedlineTA>Mol Biochem Parasitol, 2003
- We investigated a possible relation between prolactin receptor PRL R or IL 2 receptor  [ **IL2RB** ]  alpha IL 2Ralpha, CD25  [ **IL2RA** ]  expression on circulating T lymphocytes and their apoptosis in patients with breast cancer.  Ref: 12698200 Br J Cancer, 2003
- AGC10 also decreased the intracellular levels of CD25  [ **IL2RA** ] , CD122  [ **IL2RB** ] , and CD132 in CD4 and CD8 cells stimulated with the T cell mitogen phytohemagglutinin PHA.  Ref: 12467977 Mol Biochem Parasitol< MedlineTA>Mol Biochem Parasitol, 2003
- Activation markers CD25  [ **IL2RA** ] , CD122  [ **IL2RB** ] , CD69 and CD80 were also present on fetal T cells.  Ref: 12880639 Dev Comp Immunol, 2003
- We found a defective T cell response in malnourished children, as indicated by diminished levels of circulating total CD3, helper CD4, IL 2 receptor  [ **IL2RB** ]  bearing CD4 CD25  [ **IL2RA** ]  and memory helper T cell responses CD4 CD45RO in keeping with the decreased specific IgE levels against Ascaris lumbricoides.  Ref: 12848201 J Trop Pediatr, 2003
